# Comparative Magnetic Resonance Imaging and Histopathological Correlates in Two SOD1 Transgenic Mouse Models of Amyotrophic Lateral Sclerosis

**DOI:** 10.1371/journal.pone.0132159

**Published:** 2015-07-01

**Authors:** Ilaria Caron, Edoardo Micotti, Alessandra Paladini, Giuseppe Merlino, Laura Plebani, Gianluigi Forloni, Michel Modo, Caterina Bendotti

**Affiliations:** 1 Laboratory of Molecular Neurobiology, Neuroscience Department, IRCCS Istituto di Ricerche Farmacologiche “Mario Negri”, Milan, Italy; 2 Laboratory of Biology of neurodegenerative disorders, Neuroscience Department, IRCCS Istituto di Ricerche Farmacologiche “Mario Negri”, Milan, Italy; 3 McGowan Institute for Regenerative Medicine & Centre for the Neural Basis of Cognition, Departments of Radiology & Bioengineering, University of Pittsburgh, Pittsburgh, PA, United States of America; University of Sheffield, UNITED KINGDOM

## Abstract

Amyotrophic Lateral Sclerosis (ALS) is a progressive and fatal disease due to motoneuron degeneration. Magnetic resonance imaging (MRI) is becoming a promising non-invasive approach to monitor the disease course but a direct correlation with neuropathology is not feasible in human. Therefore in this study we aimed to examine MRI changes in relation to histopathology in two mouse models of ALS (C57BL6/J and 129S2/SvHsd SOD1G93A mice) with different disease onset and progression. A longitudinal in vivo analysis of T2 maps, compared to ex vivo histological changes, was performed on cranial motor nuclei. An increased T2 value was associated with a significant tissue vacuolization that occurred prior to motoneuron loss in the cranial nuclei of C57 SOD1G93A mice. Conversely, in 129Sv SOD1G93A mice, which exhibit a more severe phenotype, MRI detected a milder increase of T2 value, associated with a milder vacuolization. This suggests that alteration within brainstem nuclei is not predictive of a more severe phenotype in the SOD1G93A mouse model. Using an ex vivo paradigm, Diffusion Tensor Imaging was also applied to study white matter spinal cord degeneration. In contrast to degeneration of cranial nuclei, alterations in white matter and axons loss reflected the different disease phenotype of SOD1G93A mice. The correspondence between MRI and histology further highlights the potential of MRI to monitor progressive motoneuron and axonal degeneration non-invasively in vivo. The identification of prognostic markers of the disease nevertheless requires validation in multiple models of ALS to ensure that these are not merely model-specific. Eventually this approach has the potential to lead to the development of robust and validated non-invasive imaging biomarkers in ALS patients, which may help to monitor the efficacy of therapies.

## Introduction

Amyotrophic Lateral Sclerosis (ALS) is a progressive neurodegenerative disease characterized by the loss of upper motoneurons in the cortex, as well as lower motoneurons in the brainstem and spinal cord. In some cases, the first symptoms are misinterpreted, with the diagnosis of ALS based on clinical criteria occurring more than a year later. The heterogeneity of the clinical presentation of ALS, with respect to diversity of symptoms at onset, as well as the severity and progression of the disease, further complicates diagnosis and prognosis. Even in families with specific gene mutations, clinical heterogeneity is evident in the age and site of symptom onset, as well as disease progression [1].

Specific and sensitive biomarkers of ALS pathology using non-invasive analytical tools that could identify patients at the symptom onset are therefore essential to provide a faster diagnosis and provide a means to classify patients for clinical trials of novel therapeutics. Indeed, diagnostic imaging techniques, such as positron emission tomography, single photon emission computed tomography and magnetic resonance imaging (MRI), afford a serial assessment of pathology in the brain and the spinal cord of patients during the disease course and are hence valuable to document pathological changes [2]. The widespread availability, anatomical resolution and diversity of information obtainable by MRI position it as a promising tool to uncover region-specific biomarkers. At present, MRI is clinically mostly used for differential diagnosis to exclude other “ALS-mimicking” syndromes [3]. However, due to the heterogeneity of the clinical presentation of ALS and the diverse time courses, it is challenging to develop and validate biomarkers purely based on the patient population. The use of animal models therefore is an important investigative tool to establish and validate MRI-based biomarkers [4].

A familial form of ALS can be modeled in transgenic mice by overexpressing multiple copies of the human mutant SOD1 gene [5]. This model recapitulates several features of ALS, notably a progressive loss of lower motor neurons and axons of the ventral motor roots leading to muscle atrophy, paralysis, and eventually death [6–8]. Remarkable differences in the severity of disease phenotype have been noted in SOD1G93A mice, depending not only on transgene copy number [9], but also and in particular on their genetic background [10–12]. In fact, we observed that transgenic SOD1G93A mice in distinct inbred strains, namely C57BL/6J and 129S2/SvHsd (129SV), differed remarkably in disease progression and life span [13], as well as their response to treatment with lithium and omega-3 polyunsaturated fatty acids [14, 15]. We also identified marked differences in the motor neuron transcriptome between these two ALS mouse models, as indices of fast and slow disease progression, which may prove useful in identifying potential disease modifiers responsible for the heterogeneity of human ALS [16].

The availability of strong magnetic field has allowed the application of MRI also in rodent models of neurological disease, affording validation of MRI changes by histopathology. Indeed, MRI has been used to document brain atrophy and an increase in tissue degeneration in SOD1G93A mice [17–22]. However, it remains unclear if these changes precede or follow the loss of brainstem motoneurons and how these alterations relate to disease state [23–25].

We therefore aimed to determine the potential of multimodal MRI of the brainstem and lumbar spinal cord to discriminate between two SOD1G93A mouse models of ALS with different rate of disease progression. We identified changes in cranial nuclei (as T2 relaxation time) and in white matter spinal cord (as diffusion tensor imaging) between the two SOD1G93A mouse strains. For validation of putative MRI biomarkers, histological studies have been performed to determine the extent of nuclei degeneration and axonal disruption in these ALS mouse models.

## Materials and Methods

### Ethical statement

All the experiments and the protocol proposed in the projects were examined and approved first by Institutional Animal Care and Use Committee (IACUC) and then authorized by the Italian Ministry of Health (Decreto n 35/2012-B). Procedures involving animals and their care were conducted according to the Mario Negri’s institutional guidelines that are in compliance with national (D.L. no. 116, G.U. suppl. 40, Feb.518, 1992, Circular No.8, G.U., 14 luglio 1994) and international laws and policies (EEC Council Directive 2010/63/UE). The mice were bred and maintained in a SPF environment. Animals were monitored weekly for motor impairments and changes in body weight. Animals with motor impairment had pellet and powdered food in a petri dish on the cage bottom and water bottles with long drinking spouts. When the animals were unable to right themselves within 10 s after being placed on either side, they were humanely euthanized by deep anesthesia with Equithesin. Mice were then sacrificed by decapitation followed by the dissection of tissues or underwent intracardiac perfusion to obtain tissues for immunohistochemistry. This humane end point was defined as survival length. If animals suffered infections and showed a sudden weight loss greater than 20% in one week, they were euthanized by CO_**2**_ asphyxiation. During the entire period of MRI acquisition (45 min for each mouse), animals were maintained under gaseous anesthesia.

### Transgenic animals

Female transgenic mice modeling ALS expressed ~20 copies of the mutant human gene SOD1 with a Glycine to Alanine substitution in position 93 (SOD1G93A mice) [5] either on C57BL/6J strain (B6.Cg-Tg(SOD1*G93A) 1Gur/J, Jackson Laboratories, here indicated as C57 SOD1G93A) or on 129S2/SvHsd strain (129Sv SOD1G93A) generated in house [14]. Transgenic mice were identified by PCR performed on DNA from tail biopsies. Samples were completely digested by overnight incubation at 55°C in Direct-PCR lysis Buffer (Viagen Biotech, Los Angeles, California, USA) containing 0.1μg/μl of Proteinase K (Promega). The following day, they were incubated at 85°C for 30 minutes, to inactivate the Proteinase K, and then analyzed by PCR. 50ng of DNA from each animal were used as a substrate for qualitative PCR, in a mix containing 1x PCR buffer, GoTaq polymerase (0.25U), deoxyNTPs (250 μM each), specific forward and reverse primers (0.5μM each) in a final volume of 10μl. All reagents were purchased by Promega, except for primers that were synthesized by Life Technologies. Mice were housed under standard conditions (22 ± 1°C, 60% relative humidity, 12 hour light/dark schedule, 3–4 mice/cage, with free access to food and water). Although expressing the same amount of transgene copies, C57 and 129Sv mice display a very different disease phenotype in terms of disease onset (15.9 ±1.0 in C57 and 14.5 ±0.6 in 129Sv mice) (mean±SD) and survival (24.2±1.8 in C57 and 17.8±0.8 in 129Sv mice) (S1 Fig). The different phenotype is well described and it has previously been reported in several publications [13–15].

### Experimental Design

Animals were randomly assigned to two different experiments: 1. In vivo serial T2-weighted MRI acquisitions and 2. Ex vivo time course DTI and histopathological analyses. In both experiments, transgenic mice were compared with their respective non-transgenic (Ntg) littermates.

**Fig 1:**
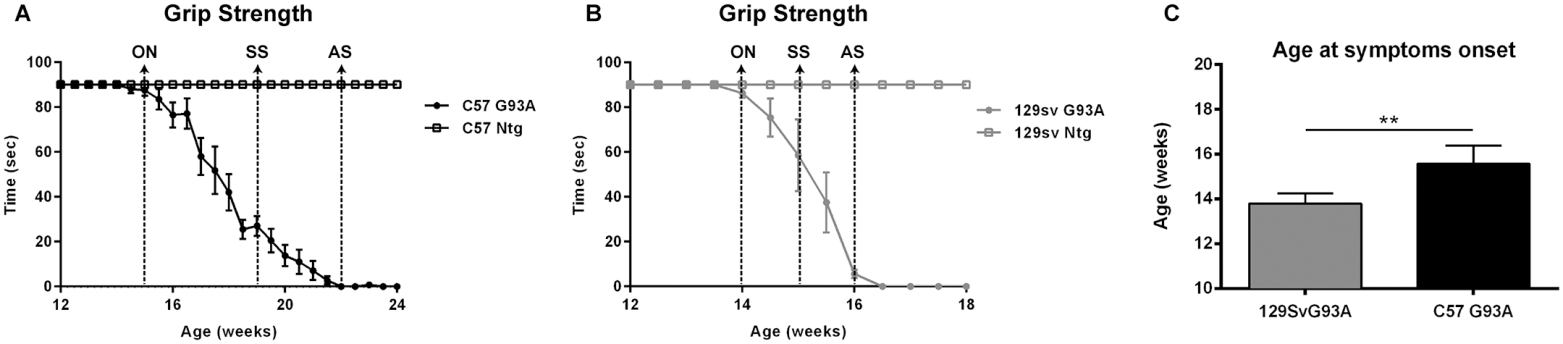
Disease progression in C57 and 129Sv SOD1G93A mice. Grip strength measurements of C57 SOD1G93A (A) and 129Sv SOD1G93A (B) mice analysed in vivo for T2 relaxation time. Time points of MRI and histological studies are reported as arrows in correspondence to the respective ages of analysis: ON = Onset, SS = symptomatic, AS = advanced stage. (C) Disease onset of C57 SOD1G93A (15.6 ± 0.8) and 129Sv SOD1G93A mice (13.8 ± 0.4) analysed with T2 MRI. Statistical analysis: Student’s t-test. ** = p-value<0.01. Data are expressed as mean ± SD. N = 6 animals for C57 SOD1G93A mice and 5 animals for 129Sv SOD1G93A mice.

Animals assigned to in vivo analysis of T2 relaxation time of brainstem were monitored for disease progression using grip strength test (see below) (Fig 1). In vivo data acquisition started at 7 weeks of age in both strains as a baseline and then followed at different time points during the disease progression. In Fig 1 the differences in the disease progression between the two strains are shown, starting from the disease onset (Fig 1C), which corresponds to the first time point at which the mice are unable to maintain the grip to the grid for at least 90 seconds. The different time points of the MRI analysis are indicated by arrows in the graphs for each of the two SOD1G93A mouse strains. For the in vivo analysis, the C57SOD1G93A mice were examined at 11, 15, 19 and 22 weeks of age corresponding to the pre-symptomatic, onset, symptomatic and advanced stage. The 129Sv SOD1G93A mice were examined only at the 11, 14 and 16 weeks of age, corresponding to pre-symptomatic, onset and advanced stage of the disease. The intermediate stage of 15 weeks was not included in this analysis to avoid subjecting the mice to frequent anaesthesia for in vivo MRI analysis.

For the histopathology of the brainstem, C57 mice were sacrificed at 11, 15, 19 and 22 weeks as representative of pre-symptomatic, onset, symptomatic and advanced stage, respectively, while 129Sv mice were examined only at 16 weeks of age when there was a small but significant effect in T2. Instead, for ex vivo DTI analyses, both strains were analysed at the onset, symptomatic and advanced stage of the disease, corresponding to 15, 19 and 22 weeks for C57 mice and 14, 15 and 16 weeks for 129Sv mice. The same tissues of the ex vivo DTI were used for the histopathology of the spinal cord and the count of axons in white matter.

### Grip strength measurement

Briefly, for the grip strength test, animals were placed on a horizontal grid at about 30 cm from the table and the tail was gently pulled until they grabbed the grid with their fore and hind paws. The grid was then gently inverted and the latency time of the mouse to fall on the table was recorded for a maximum of 90 sec. The test was repeated three time with at least 5 min of rest between each and the maximum time of latency was recorded. The onset of motor symptoms was determined by the inability of mice to complete the grip strength test in 90 sec in two consecutive sessions. By these analysis we confirmed an onset of symptoms of C57 SOD1G93A mice (n = 6) at 15.6 ±0.8 weeks of age (mean body weight 18.8g ± 1.2), while 129Sv SOD1G93A mice (n = 5) show the onset of motor impairment at 13.8± 0.4 weeks (mean body weight 21.2g ± 0,4) (Fig 1C). These data are very similar to those obtained from the overall SOD1G93A mouse strains (S1A Fig) [13–15], confirming the low variability in disease phenotype of our colonies.

### Magnetic Resonance Imaging (MRI)

MRI acquisition was performed on a 7T small bore animal Scanner (Bruker Biospec, Ettlingen, Germany), equipped with a BGA 12 gradient system (400 mT/m, rise time 110 μs). Two actively decoupled radio frequency coils were used: a volume coil of 7.2 cm diameter used as the transmitter and an anatomically shaped quadrature surface coil as the receiver.

### In vivo T2-weighted MRI acquisition

For T2-weighted analysis, C57 SOD1G93A (n = 6) and 12Sv SOD1G93A (n = 5) mice were used and compared to three respective non-transgenic littermates. Mice were anesthetized with 3–4% isoflurane (in 70:30 N_2_O:O_2_; v:v) and maintained under anesthesia with 1.0–1.5% isoflurane. Mice were secured using a head-holder with stereotaxic ear bars to reduce motion artifacts. Temperature was maintained at 37±0.5°C by a feedback controlled water circulating heating cradle. Respiratory rate was monitored to maintain the depth of anesthesia during the MRI scanning. Sagittal T2 maps of SOD1G93A and Ntg mice were acquired. For anatomical orientation, 6 axial and 10 coronal T2-weighted slices were acquired using a spin echo sequence with rapid acquisition with refocused echoes (RARE, TR 2000 ms, TE 12 ms, 1 average, field of view (FOV) 20 x 20 mm with 256 x 256 matrix and slice thickness of 1 mm). To generate sagittal T2 maps, a 20 echo train (ET) was acquired with the following parameters: TR 2500 ms, initial TE 11 ms, TE increment 11 ms, FOV 20 x 15 mm with 256 x 198 matrix and slice thickness of 0.8 mm, 45 minutes scanning time.

### T2 relaxation measurements

**Fig 2:**
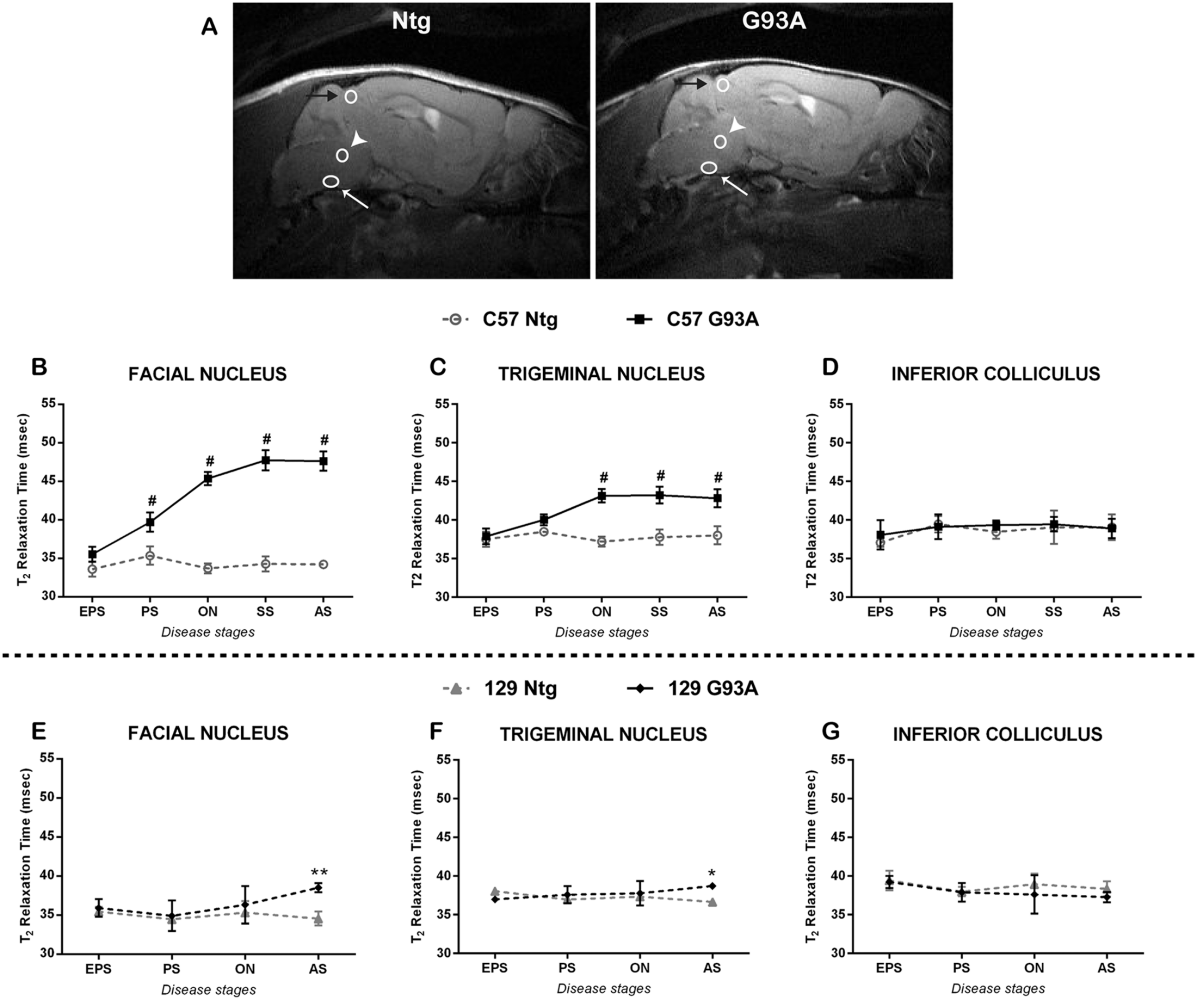
T2 relaxation time of brainstem nuclei of SOD1G93A mice at different stages of the disease. (A) Representative T2-weighted images are shown from a SOD1G93A (G93A) and a non-transgenic (Ntg) mouse. The ROIs correspondent to facial nuclei are indicated by a white arrow, to the trigeminal nuclei are indicated by a white arrowhead and to the inferior collliculi are indicated by a black arrow. T2 relaxation times of facial (B, E) and trigeminal (C, F) nuclei and of inferior colliculus (D, G) have been analyzed in C57 (B, C, D) and 129Sv (E, F, G) mice. SOD1G93A and non-transgenic mice have been compared during the progression of the disease for each strain. EPS = early pre-symptomatic stage, PS = pre-symptomatic stage, ON = Onset, SS = Symptomatic stage, AS = advanced stage. Data are expressed as mean ± SD. Statistical analysis: Two-way ANOVA, followed by Bonferroni’s post-hoc test. Facial nucleus: in C57 mice genotype [F (1,7) = 623.8, p<0.0001], stage [F (4,28) = 52.82, p<0.0001] and interaction [F (4,28) = 52.86, p<0.0001], while in 129Sv genotype [F (1,6) = 14.52, p = 0.0089], stage [F (3,18) = 2.481, p = 0.0939] and interaction [F (3,18) = 2.869, p0.0652]. Trigeminal nucleus: in C57 mice genotype [F (1,7) = 72.81, p<0.0001], stage [F (4,28) = 15.99, p<0.0001] and interaction [F (4,28) = 18, p<0.0001]. Inferior Colliculus: genotype [F (1,7) = 0.5181, p = 0.495], stage [F (4,28) = 2.329, p = 0.0806] and interaction [F (4,28) = 0.4251, p = 0.7892], while in 129Sv genotype [F (1,6) = 4.081, p = 0.0899], stage [F (3,18) = 0.5146, p = 0.6774] and interaction [F (3,18) = 3.294, p = 0.0443]. Stars indicate: * = p-value<0.05, ** = p-value<0.01, # = p-value<0.001. 6–5 animals in the SOD1G93A and 3 animals in the Ntg groups.

For the analysis and the quantification of T2 relaxation time in brainstem nuclei, the free software ImageJ has been used. T2 maps were generated by linear fitting of the decay curve of 20 echoes. Regions of Interest (ROIs) were drawn corresponding to the facial nucleus, the trigeminal nucleus and the inferior colliculus, according to a reference atlas (Allen brain Atlas) (Fig 2A). The exact localization of trigeminal and facial nuclei was determined in a mouse at the advanced stage that showed a clear hyperintensity in both nuclei. This allowed defining and fixing the distance between trigeminus and facialis in order to identify a combined ROI. Then, for each mouse the combined ROI was adjusted in correspondence to the facial nucleus (easy to recognize due its location in the ventral part of the hindbrain, between the caudal pons and the rostral medulla) followed by the identification of the trigeminal ROI. The ROI inferior colliculus, situated in the dorsal part of the brain (between the cerebellum and the cortex), was used as a negative control region.

### Ex vivo Diffusion Tensor Imaging (DTI) acquisition

In vivo DTI of a mouse spinal cord remains challenging due to prohibitive acquisition times, as well as movement artefacts. To afford an adequate acquisition DTI was therefore acquired ex vivo. Diffusion anisotropy has been demonstrated to be unaffected by fixation and preserve inherent regional characteristics [26].

For DTI analysis, four animals per group at each stage were analyzed. Mice were perfused with ice cold 0.1M PBS followed by 4% paraformaldehyde. To preserve the natural shape and morphology of the spinal cord, the cords were kept inside the vertebral columns and were immersed in the same fixative overnight at 4°C, then transferred to PBS. Before MRI imaging, each spinal cord was covered with a plastic wrap and then embedded in agarose gelatin (1:5 in H_2_O) within a 2.5 cm diameter plastic MR-compatible test tube. The surface coil was placed over the lumbar tract of the spinal cord. Slices for DTI (Spin Echo, TR = 5000 ms, TE = 38.4 ms, 1 Average, 5 B0, 30 directions with b-value = 800 s/mm^2^, δ = 12 ms, Δ = 22ms, FOV 20x20mm, 256x256 matrix, in plane resolution 0.078 mm, 10 coronal slices at 1 mm thickness, 12.5h) were aligned perpendicularly to the spinal cord axis, with the first slice at the intersection of the vertebral column and the second last rib.

### DTI image analysis

After the ex vivo acquisition of the lumbar spinal cord by DTI, specific ROI were traced in order to surround the whole section or specific regions of the spinal cord on trace weighted images. Fractional anisotropy (FA), axial diffusivity and radial diffusivity were calculated using MedInria (Asclepios Research Project—Inria Sophia Antipolis, http://www-sop.inria.fr/asclepios/). ImageJ (http://rsbweb.nih.gov/ij/) was used to trace free hand ROIs in the white matter: dorsal white matter (dWM), dorso-lateral white matter (dlWM), ventro-lateral white matter (vlWM) and ventro-medial white matter (vmWM).

### Histopathological analyses

For histopathological analyses, parallel groups of SOD1G93A mice were sacrificed at different disease stages corresponding to those examined by MRI. Mice were transcardially perfused with 50 ml of 0.1M PBS followed by 50 ml of 4% paraformaldehyde. As previously described [14], brains and spinal cords were serially sectioned on a cryostat at -20°C (20μm) in a coronal plane. Every fourth brain section was processed with Cresyl Violet (Nissl staining) for neuronal counting. For each group (n = 4), 9 and 12 sections were analyzed for trigeminal or facial nucleus, respectively.

Adjacent sections were stained with Haematoxylin and Eosin (H&E) for a better qualitative assessment of the tissue vacuolization.

### Nissl staining

For Nissl staining, slides were de-hydrated and re-hydrated in a series of alcohols. Slides were then immersed in cresyl-violet 0.5% solution in water for 3 min, washed and moved to 96% ethanol solution. Slides were then dipped for 1 min in 3% acetic acid solution in ethanol to remove the excess of Cresyl Violet. Then slides were placed in absolute ethanol and in xylene, before being coverslipped with DPX mounting medium (BDH, Poole Dorset, UK). Nissl stained samples were acquired at a 10x magnification under an Olympus BX61 light microscope and images of the trigeminal and facial nuclei were collected with a camera, using the AnaliSYS software (Soft Imaging Systems, ver. 3.2). Clear stained motor neurons, displaying a dark nucleolus and a clear nucleus, were counted using ImageJ. Since we count neurons every forth sections, the final counts were then multiplied by four, in order to estimate roughly the total number of neurons in the nuclei, as reported by Haenggeli et al. [23]. Four mice per group were analyzed.

### Haematoxylin & Eosin (H&E) staining

Since it was difficult to clearly identify the vacuolated parenchyma around neurons with Nissl staining, we decided to perform the H&E staining on adjacent sections. Briefly, sections were dipped in Hematoxylin solution (1% Hematoxylin, 5% aluminum potassium sulfate, 0.02% sodium iodate, 5% chloral hydrate and 1% citric acid, in distilled water) for 3 minutes. After a wash in running tap water, sections were placed in Eosin solution (Eosin 1% in distilled water) for 1 minute. Sections were then washed in running tap water and dehydrated through a graded series of ethanol. Finally, the sections were fixed in Xylene before being coverslipped using DPX mountant (BDH, Poole Dorset, UK). Slides were examined by an operator unaware of the animal group in order to give a qualitative evaluation of the presence or not of vacuoles in all sections. Four mice per group were analyzed.

### SMI-31 immunohistochemistry

Coronal sections of the L2 segment of the spinal cord were stained with SMI-31 marker, which allows the detection of phosphorylated neurofilaments that compose axons and is therefore considered a marker of axonal integrity. Free-floating sections were first treated with H_2_O_2_ 1% in PBS 0.01 M for 10 minutes to inhibit endogenous peroxidases. Then, they were incubated for 1 h at room temperature (RT) in a blocking solution containing normal goat serum 10% in PBS. Samples were incubated overnight at 4°C with the primary monoclonal antibody against SMI-31 [1:7500] (Covance), diluted in PBS containing normal serum 1%. The day after, sections were probed with the corresponding secondary biotinylated antibody (1:200, Vector Labs) in 1% normal serum and PBS for 1 hour. After 3 washes, sections were immersed in avidin-Biotin peroxidase solution (Vectastain kit, Vectro labs) for 1 hour. Immunohistochemistry was revealed by the reaction with 3’-3-diaminobenzidine tetrahydrochloride (DAB, 0.5 mg/ml in TBS + hydrogen peroxide at a final concentration of 0.009%). Subsequently sections were washed, mounted on gelatine-covered glass slides and dried overnight at room temperature. The day after sections were dehydrated through a graded series of alcohols, fixed in Xylene and cover slipped using DPX mountant (BDH, Poole Dorset, UK). SMI-31 stained sections were acquired at a 40x magnification under an Olympus BX61 light microscope.

### Axons quantification

Axons of the L2 segment of the lumbar spinal cord were counted at a magnification of 40x, in serial sections, using the free software ImageJ. 3 animals per each group have been analysed. As SMI-31 positive axons were readily identified, it was possible to isolate these using a threshold based on their higher grey values. Using the free software ImageJ, each axon separately was counted using the command ‘Analyse particles’. For each animal, we analysed two 30μm thick sections, sampled in the L2 segment and distanced one from another 600 μm. The count of axons of the two sections was averaged and the resulting number was used for statistical analysis.

### Statistical Analysis

The analysis of MRI images and the quantification of neurons, vacuoles and white matter axons count were performed blinded. For the longitudinal measure of T2 and for the analysis of ex-vivo DTI parameters a two-way ANOVA followed by Bonferroni’s post-hoc test was applied for the comparison between Ntg and SOD1G93A mice. For the motor neurons count, a two-way ANOVA or Student’s t-test was used as appropriate.

## Results

### Analysis of cranial nuclei degeneration

C57 SOD1G93A mice developed a delayed onset of motor symptoms and a slower disease progression compared to 129Sv mice despite expressing the same amount of human SOD1 transgene products. In fact, disease duration was more than doubled in C57 SOD1G93A with respect to 129Sv mice (8.3 ±2.1 vs 3.2 ± 0.6 weeks, respectively) (Fig 1).

Serial in vivo MRI was applied to sagittal sections of brain from the two ALS mouse models at different times during the course of the disease to examine signal changes inside different nuclei of the brainstem as potential markers of disease onset and progression (Fig 2). In C57 SOD1G93A mice the T2 relaxation time in the facial nucleus (Fig 2B) and trigeminal nucleus (Fig 2C) was significantly higher compared to non-transgenic littermates. Even in the pre-symptomatic stage, a significant difference in the facial nucleus was already evident and progressively increased during the disease course. In contrast, the trigeminal nuclei exhibited the first significant increase concomitant with the onset of motor symptoms. Interestingly, this signal change did not further increase with disease progression. Conversely, the 129Sv SOD1G93A mice showed no differences in T2 relaxation time of the cranial nuclei until the advanced stage of the disease (Fig 2E and 2F). However, even at this stage, the T2 increase in the facial and trigeminal nuclei was much lower compared to C57 SOD1G93A mice. These changes were nuclei specific, as no change in T2 was evident in the inferior colliculus (Fig 2D and 2G), which served as an internal control in this study, since it is an unaffected region.

**Fig 3:**
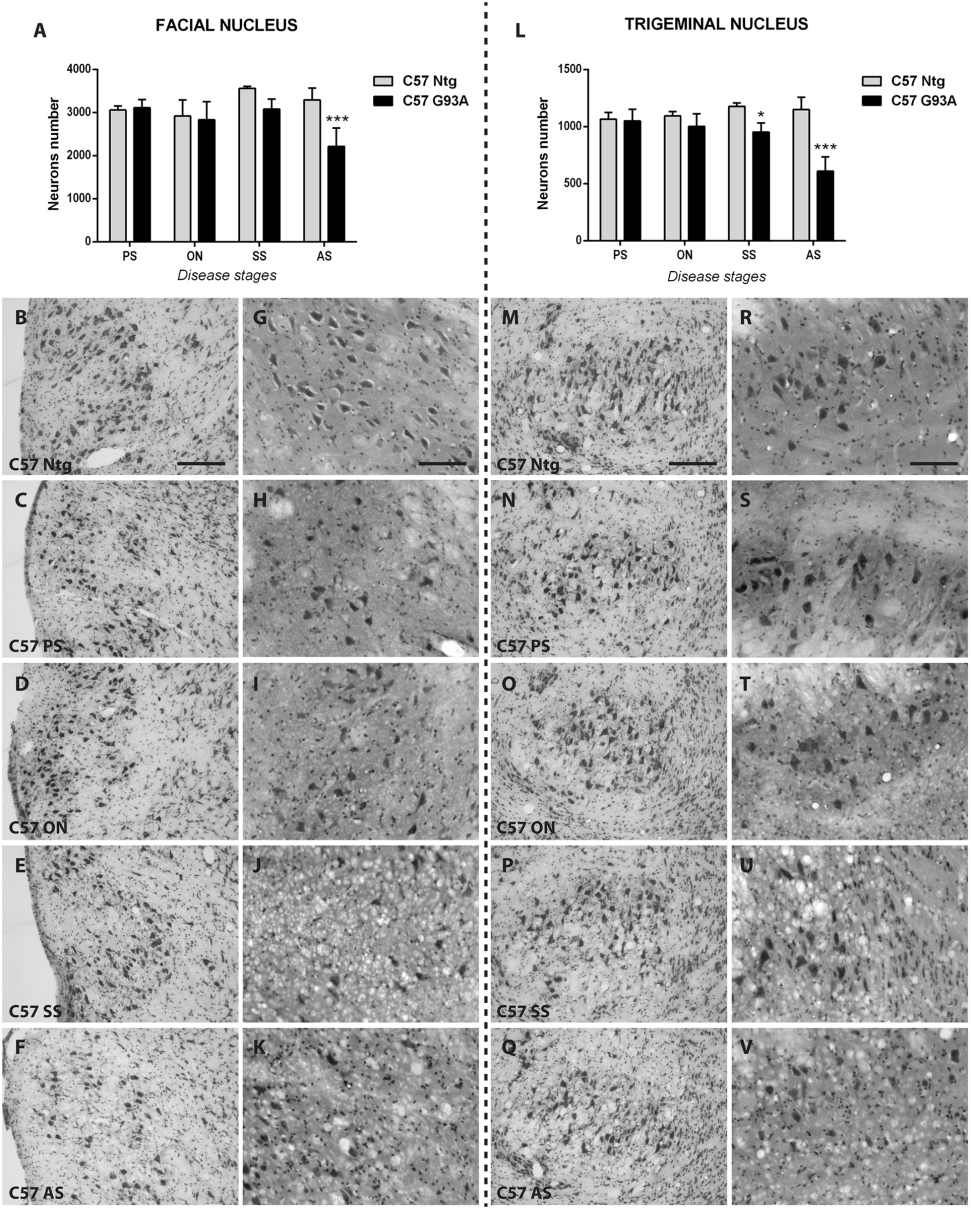
Evaluation of cranial neuron number and their vacuolization in C57 SOD1G93A mice and non-transgenic littermates. Facial (A-K) and trigeminal (L-V) nuclei have been analyzed by Nissl (B-F, M-Q) and H&E (G-K, R-V) staining to quantify brainstem neurons and to detect vacuolization, respectively. (A) Neuronal cell count of facial nucleus of C57 SOD1G93A mice at different disease stages and relative non-transgenic littermates. (B-F) Nissl staining microphotographs of the facial nucleus of non-transgenic (Ntg, B) and SOD1G93A (C-F) C57 mice at pre-symptomatic (C), onset (D), symptomatic (E) and advanced stage (F) of the disease. G-K: H&E staining microphotographs of the facial nucleus of non-transgenic (Ntg, G) and SOD1G93A (H-K) C57 mice at pre-symptomatic (H), onset (I), symptomatic (J) and advanced stage (K) of the disease. (L) Neuronal cell count of trigeminal nucleus of C57 SOD1G93A mice at different disease stages and relative non-transgenic littermates. M-Q: Nissl staining microphotographs of the trigeminal nucleus of non-transgenic (Ntg, M) and SOD1G93A (N-Q) C57 mice at pre-symptomatic (N), onset (O), symptomatic (P) and advanced stage (Q) of the disease. (R-V) H&E staining microphotographs of the trigeminal nucleus of non-transgenic (Ntg, R) and SOD1G93A (S-V) C57 mice at pre-symptomatic (S), onset (T), symptomatic (U) and advanced stage (V) of the disease. Scale bars represent 100 μm in Nissl microphotographs (B-F, M-Q) and 200 μm in H&E microphotographs (G-K, R-V). PS = pre-symptomatic stage, SS = Symptomatic stage, AS = advanced stage. The total neurons number was obtained multiplying the raw counts by four to give an estimate of the total cell number in the nucleus. Data are expressed as mean ± SD. Statistical analysis: Two-way ANOVA, followed by Bonferroni’s post-hoc test. Statistical analyses revealed, for the facial nucleus, significant effects for genotype [F (1,6) = 26.59, p = 0.0021], for stage [F (3,18) = 4.486, p = 0.0161] and interaction [F (3,18) = 6.298, p = 0.0041]. For the trigeminal nucleus, significant effects were revealed for genotype [F (1,6) = 152.8, p<0.0001], for stage [F (3,18) = 8.251, p = 0.0012] and interaction [F (3,18) = 14.13, p<0.0001]. Stars indicate: * p-value<0.05, *** p-value<0,001 between SOD1G93A and respective Ntg littermates. 4 animals for each group.

The neuron counts in both cranial nuclei of C57 SOD1G93A mice exhibited no differences with respect to non-transgenic littermates at the pre-symptomatic and onset stages of the disease. The first sign of neuronal loss emerged at the symptomatic stage only in the trigeminus (19% reduction) while in the facial nucleus there was only a trend (14% reduction, p>0.05). At the advanced stage of the disease, a significant 37% and 47% decrease of neurons was detected for facial and trigeminal nuclei, respectively (Fig 3A and 3L). Nevertheless, structural changes in the parenchyma around motor neurons were observed in C57 SOD1G93A mice at an earlier stage compared to the Ntg littermates (Fig 3G and 3R). In particular, at the pre-symptomatic and onset stage of the disease, small vacuoles in facial and trigeminal nuclei were observed (Fig 3H-3I and 3T) despite no motor neuron loss. These vacuoles further increased with disease progression. At the symptomatic (Fig 3J and 3U) and advanced stage (Fig 3K and 3V), the parenchyma in both facial and trigeminal nuclei was covered by numerous large vacuoles.

**Fig 4:**
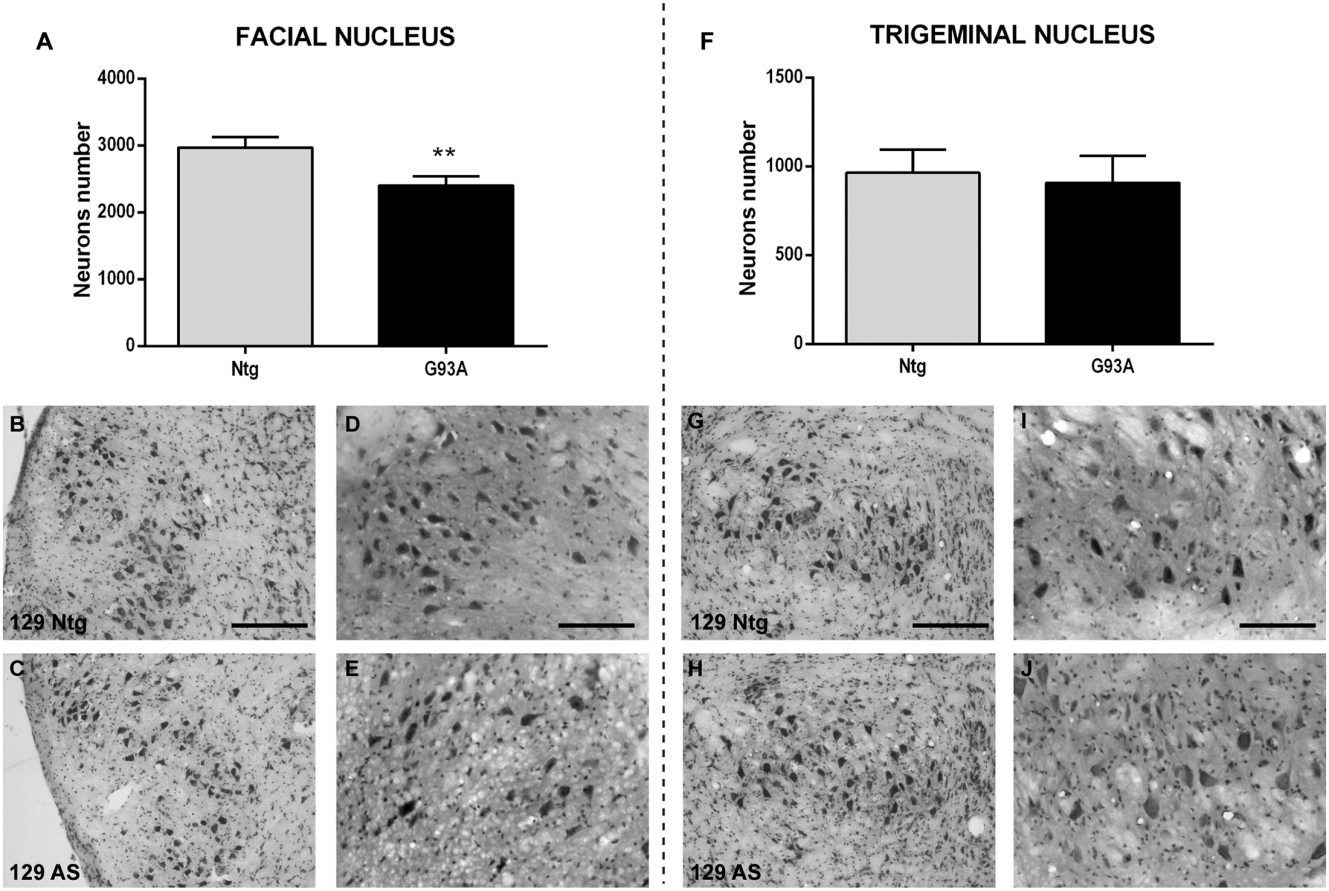
Evaluation of cranial neuron number and their vacuolization in 129Sv SOD1G93A mice and non-transgenic littermates. Facial (A-E) and trigeminal (F-J) nuclei have been analyzed by Nissl (B-C, G-H) and H&E (D-E, I-J) staining to quantify brainstem neurons and to detect vacuolization, respectively. (A) Neuronal cell count of facial nucleus of 129Sv SOD1G93A mice at the advanced stage of the disease and relative non-transgenic littermates. (B-C) Nissl staining microphotographs of the facial nucleus of non-transgenic (Ntg, B) and SOD1G93A (C) 129Sv mice at the advanced stage of the disease. (D-E) H&E staining microphotographs of the facial nucleus of non-transgenic (Ntg, D) and SOD1G93A (E) 129Sv mice at the advanced stage of the disease. (F) Neuronal cell count of trigeminal nucleus of 129Sv SOD1G93A mice at the advanced stage of the disease and relative non-transgenic littermates. (G-H) Nissl staining microphotographs of the trigeminal nucleus of non-transgenic (Ntg, G) and SOD1G93A (H) 129Sv mice at the advanced stage of the disease. (I-J) H&E staining microphotographs of the trigeminal nucleus of non-transgenic (Ntg, I) and SOD1G93A (J) 129Sv mice at the advanced stage of the disease. Scale bar represents 100 μm in Nissl microphotographs (B-C, G-H) and 200 μm in H&E microphotographs (D-E, I-J). AS = advanced stage. The total neuron number was obtained multiplying the raw counts by four to give an estimate of the total cell number in the nucleus. Data are expressed as mean ± SD. Statistical analysis: Student’s t-test. ** p-value<0.01 between SOD1G93A and respective Ntg littermates. 4 animals for each group.

Conversely, the histopathological analysis of 129Sv SOD1G93A mice, at the time in which the T2 increase was observed (advanced stage, Fig 2E and 2F), revealed a slight decrease (20%, p = 0.0022) of the neurons count of the facial nucleus, while the trigeminus did not show neuronal loss even at the advanced stage of the disease (p = 0.86, Fig 4A and 4F). At this stage some extracellular vacuoles were present in the cranial nuclei of 129Sv SOD1G93A mice (Fig 4E and 4J), compared to Ntg littermates (Fig 4D and 4I). However, the vacuolization appeared at a much lower degree in the trigeminus nucleus than in the facial nucleus, which is in accordance with the milder alteration in the T2 relaxation time found in the trigeminus.

### White matter (WM) degeneration in spinal cord

**Fig 5:**
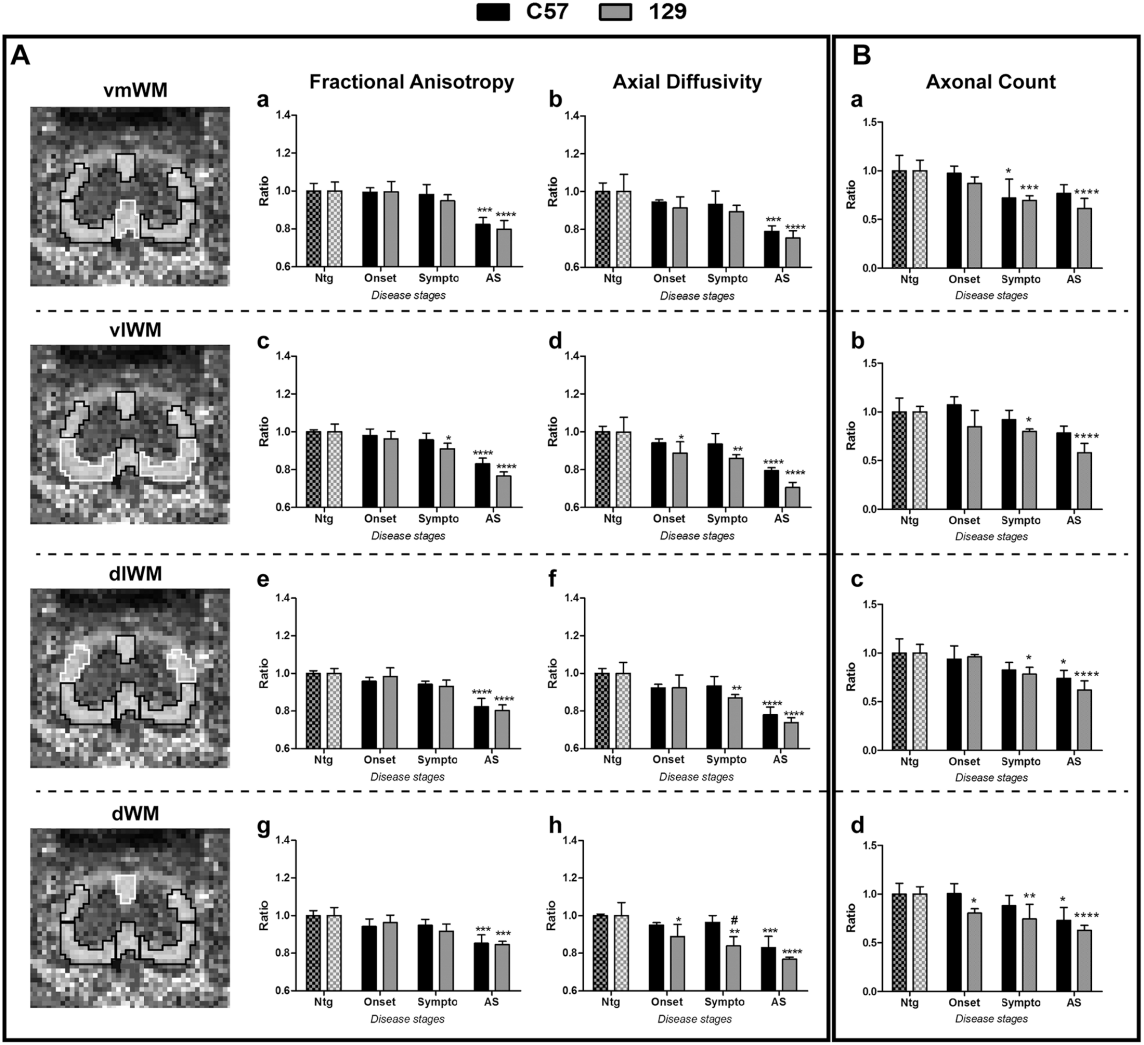
Diffusion Tensor Imaging (DTI) parameters and axonal count of lumbar spinal cord in C57 and 129Sv SOD1G93A mice during disease progression. (A) On the left, representative images of the Fractional Anisotropy of the lumbar spinal cord have been reported. Different white matter regions were took into consideration for the analyses: ventro-medial (vmWM), ventro-lateral (vlWM), dorso-lateral (dlWM) and dorsal (dWM). White lines represent the white matter area taken into consideration. Different diffusion parameters have been considered for the analysis of white matter degeneration through DTI: Fractional Anisotropy (a,c,e,g) and axial diffusivity (b,d,f,h). (B) The axonal count has been performed in the same white matter regions analysed with DTI. SOD1G93A mice have been analyzed at the onset, symptomatic (Sympto) and advanced stage (AS) of the disease. Data are expressed as ratio between SOD1G93A and their respective Ntg mice. Mean ± SD is reported. Statistical analysis: Two-way ANOVA has been used to compare Ntg and SOD1G93A mice during the disease progression, followed by Bonferroni’s post-hoc test. Stars indicate: * p-value<0.05, ** p-value<0.01, *** p-value<0.001, **** p-value<0.0001, between SOD1G93A and their respective Ntg controls of the same mouse strain. # indicates p-value<0.05 between C57 and 129Sv mice of the same disease stage. 4–3 animals for each group.

The lumbar portion of the spinal cord was examined for WM degeneration by ex vivo diffusion tensor imaging (DTI) (Fig 5). Spinal cords of Ntg mice revealed no signal changes between different time points (data not shown). In contrast, both C57 SOD1G93A and 129Sv SOD1G93A mice exhibited a progressive decrease of FA and axial diffusivity over time (Fig 5A), whereas radial diffusivity increased (S2 Fig). While overall DTI alterations of the WM were evident at the advanced stage of the disease in both C57 SOD1G93A and 129Sv SOD1G93A compared to respective non transgenic mice, differences were observed between the two mouse models at earlier disease stages. In fact, a decreased axial diffusivity was detected in the ventro-lateral and dorsal WM regions of 129Sv SOD1G93A mice with respect to non transgenic mice, already at the onset of the disease (Fig 5Ad and 5Ah) while in C57 SOD1G93A mice this phenomenon was evident only at the advanced stage. The decrease of axial diffusivity partially overlapped with the reduction in the axons count of the spinal cord white matter in the two SOD1G93A mouse strains. In fact, with the exception of the ventro medial region in which the reduction of axons found in both SOD1G93A mouse strains at the symptomatic stage did not reflect a reduction in axial diffusivity, in all the other white matter regions of 129Sv SOD1G93A mice at symptomatic and advanced stage of the disease, the variations of the two parameters overlapped (Fig 5A and 5B). A massive loss of SMI31 positive fibers was evident overall in the white matter regions of both SOD1G93A mouse strain at the advanced disease stages (Fig 5B, S3 and S4 Figs), where the axonal loss led to an increased extracellular and inter-axonal spacing (S3 and S4 Figs).

## Discussion

In this work, we evaluated the significance of MRI technique in detecting and predicting differences in two familial ALS mouse models with a different disease course. Indeed, despite these mice expressing the same amount of SOD1G93A transgene and protein, their different genetic background, C57 or 129Sv, influences both the onset of motor symptoms and the rate of disease progression.

We compared the cranial nuclei degeneration between these two fALS mouse models during the disease course. This comparative analysis showed a different vulnerability of the cranial motoneurons in the two ALS animal models. However, surprisingly, the 129Sv SOD1G93A mice, with a faster disease progression, exhibited a remarkable delay in the alterations of these nuclei by both MRI and histopathological analysis, compared to C57 SOD1G93A mice. In C57 SOD1G93A mice, the MRI analysis revealed an early and progressive increase of T2 relaxation time of both facial and trigeminal nuclei, starting from the pre-symptomatic stage of the disease, as reported also by Evans et al. [22]. We demonstrated that this MRI alteration is mostly associated with the formation of vacuoles, rather than motoneurons loss. Indeed, while T2 started to increase at the pre-symptomatic stage, when also vacuolization was detected, motoneurons loss was found only at a symptomatic stage of the disease, confirming previous results [19, 23–25, 27, 28]. Our results corroborate data obtained in previous longitudinal studies, where SOD1G93A mice displayed a significant higher T2 value in cranial nuclei, compared to non-transgenic littermates [17, 20–22, 29]. The correspondence that we detected between a higher T2 relaxation time and the vacuoles formation is in line with previous results obtained by Bucher and co-workers [20]. Moreover, we demonstrated, for the first time, that these signal alterations precede motoneuron loss, which occurs only later in the disease course. However, the recent paper from Evans et al. questioned a causal relationship between vacuolization and T2 increase, suggesting that also an increased astrocytosis and microgliosis correlated to a similar extent with T2 intensity [22]. Since in the present study we have not examined astroglia and microglia in the brainstem of the two SOD1G93A mouse strains, we cannot exclude that the differences found in T2 intensity in both facial and trigeminal nuclei may depend on different reactive glial responses between the two mouse strains. Nevertheless, vacuole formation remains one of the earliest signs of motor neuron alterations. In fact, vacuolization, which is primarily due to abnormal and swollen mitochondria, has been observed as early as one month of age in dendrites and proximal axons of spinal motoneurons [30, 31], before the loss of motor neurons perikaria and the appearance of a glial reaction [6]. Here we reported a clear vacuolization at the level of neuropil of cranial motor nuclei of C57 SOD1G93A mice already at the pre-symptomatic stage and in particular at the onset of motor symptoms and later on. As for the spinal motor neurons, the presence of vacuoles in the cranial motor nuclei, detected by electron and light microscopy [20, 32], does not depend on, but precedes, the motor neuron loss. However, even at a pre-symptomatic stage, i.e. asymptomatic for hind limb motor dysfunction, preliminary signs of dysfunction can be appreciated by measuring licking and mastication rates [33]. For this reason, a method such as in vivo MRI, that can visualize non-invasively the tissue degeneration in cranial nuclei, could be useful for the monitoring of motor function impairment associated with these nuclei. With respect to this, our T2 analysis is in accordance with the results presented by Evans and colleagues [22]. Indeed, our results confirm the high sensitivity and specificity of T2 relaxation time in detecting abnormalities even before motoneurons loss, supporting T2 imaging as an early biomarker of the disease. The fact that we observed a higher increase of T2 relaxation time in facial rather than in trigeminal nucleus, is also in line with that reported by Evans and co-workers [22] and can be due to the lower thickness of the latter nucleus (~0.5 mm, compared to ~1 mm of the facial nucleus). This might have influenced the level of signal acquired from 0.8 mm thick slices, i.e. the signal from the trigeminal nucleus could be affected by partial volume effects.

An interesting, if surprising, result of this study is the fact that the 129Sv SOD1G93A mice, with a faster disease progression, exhibited a remarkable delay in the alterations of the cranial nuclei detected by both MRI and histopathological analysis, as compared to C57 SOD1G93A mice. In fact, this suggests that the degeneration of brainstem nuclei is not predictive of a more severe phenotype in the SOD1G93A mouse models. Recently, the comparative analysis of the spinal cord and motor neurons of these two SOD1G93A mouse strains highlighted differences associated with the disease severity which were unrelated to the degree of motor neuron loss [13]. In fact, the loss of motor neurons was similar two weeks after the symptom onset in both the slow and fast progressing ALS mouse model. However, an earlier accumulation of ubiquitinated protein aggregates was found in the motor neurons of the 129Sv SOD1G93A compared to C57 SOD1G93A mice clearly indicating a dysfunction of remaining motor neurons [13]. A differential vulnerability of cranial motoneurons among different MND animal models has already been reported [23], suggesting that there is a selective motoneuron loss in this region which depends upon the particular mouse model and disease stage. However, since 129Sv SOD1G93A mice die much earlier than the C57 SOD1G93A mice we cannot exclude that the preservation of their brainstem motor neurons is only due to the premature death of these mice rather than their different vulnerability.

Besides brainstem degeneration, axonal loss and dysfunction are also hallmarks of ALS. Whereas the loss of motor neurons perikarya leads to morphological alterations in the grey matter, the consequent loss of axons results in the degeneration of the white matter.

Conversely to the degeneration of cranial nuclei, the alterations of the axonal pathways, detected in spinal cord white matter by DTI analysis, changes in relation to the different disease phenotype of SOD1G93A mice. In fact, the 129Sv SOD1G93A mice exhibited a lower axial diffusivity in both ventrolateral and dorsal WM regions, already at the onset of the disease, while the same parameter in C57 SOD1G93A mice decreased only at the advanced stage with reference to respective non transgenic mice. Such difference was confirmed by histological ex vivo analysis, as demonstrated by the reduced SMI-31 positive axon density in these regions of 129Sv SOD1G93A mice at the disease onset. It should be noted that these regions are crossed by the axons of spinal and cortical motor neurons, respectively. We recently demonstrated that the different phenotype of the these two SOD1G93A mice strains is not due to a different motor neuron loss in the lumbar spinal cord but rather to a difference in their function, i.e. ubiquitinated aggregates accumulation [13]. With the present study, we demonstrate that the two mouse strains remarkably differ in their axonal degeneration as demonstrated by both DTI and histopathological analysis of the white matter. Interestingly, our recent studies on the extent of muscle denervation of these two mouse models support this observation (manuscript submitted). Therefore, we hypothesize that a decreased axial diffusivity by DTI analysis can be a good indicator of an axonal damage at early disease stages [34, 35]. This is in line with the observations reported by different studies [34, 36–38].

A worsening of these alterations was found at the advanced stage, when both FA and axial diffusivity further decreased, mirroring a further axonal loss and degeneration. Moreover, at this stage, an increased radial diffusivity was observed (S2 Fig). This is probably due to the increased inter axonal space associated to the reduced axonal density and/or function as indicated by the reduction of SMI-31 staining [34, 39, 40]. Interestingly, we recently demonstrated, through the analysis of the transcriptional profile of the laser-dissected motor neurons of 129Sv SOD1G93A mice at the disease onset, a marked down regulation of specific pathways involved in the axonal transport mechanisms, i.e. neuron projection, microtubule and protein transport categories, as an index of a prominent axonal dysfunction which was not detected in C57 SOD1G93A mice at the same disease stage [16]. This supports the hypothesis that intrinsic axonal mechanisms actively determine the phenotype in ALS (reviewed in [41]).

Recently, Evans et al. reported a diffusion analysis only of the brainstem region, reporting a lower capacity of apparent diffusion coefficient (ADC) in differentiating between SOD1G93A and Ntg mice, compared to T2 analysis [22]. Differently, analyzing the lumbar spinal cord by DTI, we showed that this MRI technique has the potential to discriminate between different disease severities, being more predictive of a severe disease phenotype in SOD1G93A mice than T2 relaxation time of brainstem nuclei. Thus, we propose spinal cord DTI as a useful marker to detect early alterations in the function of motor axons with respect to the motor phenotype of SOD1G93A mice even before the loss of motor neurons.

In ALS patients, the vast majority of studies analyzed the cortico spinal tract in the brain and reported a decreased FA and an increased Mean Diffusivity in this area. However, the analysis of axial diffusivity is not always consistent, since some groups reported an increase [42], some groups a decrease [37] and some groups a lack of changes [43–45]. In contrast, papers reporting an analysis of ALS patients’ spinal cord consistently report decreased FA in the white matter of the cervical tract [46, 47] with an increased radial diffusivity [48]. These data are in line with our observations of spinal cord of SOD1G93A mice at the advanced disease stage although a reduced FA was detectable also at the symptomatic stage in fast progressing mice.

In conclusion, our study demonstrates a differential regional motoneuron vulnerability between two familial ALS mouse models. Surprisingly, the 129Sv SOD1G93A strain, although having a more severe phenotype, displayed a delayed degeneration of cranial nuclei as compared to the slowly progressing diseased mice confirming the later vulnerability of the brainstem motor neurons in these ALS mouse models. Moreover, we demonstrated that DTI analysis may predict the severity of disease progression in ALS mice. This suggestion should however be taken with caution as further studies are needed to confirm this interpretation of data in order to propose the use of axial diffusivity to predict the disease severity.

The present study underlines, once again, the importance of the comparison between the two mouse models in defining the molecular mechanisms that might eventually enable the identification of prognostic markers of the disease and afford the design of targeted therapeutic strategies for individual patients.

## Supporting Information

S1 Fig Disease phenotype of C57 and 129Sv SOD1G93A mice. Kaplan Meier plots showing the proportion of transgenic mice without symptoms of disease onset (a) and percentage of surviving animals (b) are reported. From graphs it is possible to appreciate the earlier onset and the faster disease progression in 129Sv SOD1G93A mice, compared to C57 SOD1G93A mice. Indeed, C57 SOD1G93A mice displayed first motor symptoms at 15.9 ±1 weeks of age (mean body weight 20.5g ± 1.7) and died at 24.5 ±1.6 weeks (mean body weight 18g ± 1.7), while 129Sv SOD1G93A mice show the onset of motor impairment at 14.5± 0.6 weeks (mean body weight 17.2g ± 2.5) and they died at 17.8 ±0.8 weeks of age (mean body weight 14.1g ± 2.2). The statistical analysis by the Log-rank test reveals a p-value < 0.0001 for both onset and survival. (c) Weight change of C57 and 129Sv SOD1G93A mice during the disease progressio. Data are expressed as mean ± SD. N = 15 animals for C57 SOD1G93A mice and 24 animals for 129Sv SOD1G93A mice.
(TIF)

S2 Fig Radial Diffusivity of lumbar spinal cord in C57 and 129Sv SOD1G93A mice during disease progression. Radial diffusivity of different white matter regions has been reported: ventro-medial (vmWM, a), ventro-lateral (vlWM, b), dorso-lateral (dlWM, c) and dorsal (dWM, d). On the left, representative images of the Fractional Anisotropy of the lumbar spinal cord have been reported. The white line represents the white matter area taken into consideration.
(TIF)

S3 Fig Axonal degeneration in the white matter spinal cord of C57 SOD1G93A mice. Microphotographs of SMI-31 staining of the lumbar white matter spinal cord are reported from a non-transgenic (A-D) and C57 SOD1G93A mice at the onset (E-H), symptomatic (I-L) and advanced stage (M-P) of the disease. In the first line boxes, a schematic representation of the L2 coronal section is shown, with a grey square showing the white matter portion analysed: vmWM (A, E, I, M), vlWM (B, F, J, N), dlWM (C, G, K, O) and dWM (D, H, L, P). Scale bar 50 μm.
(TIF)

S4 Fig Axonal degeneration in the white matter spinal cord of 129Sv SOD1G93A mice. Microphotographs of SMI-31 staining of the lumbar white matter are reported from a non-transgenic (A-D) and 129Sv SOD1G93A mice at the onset (E-H), symptomatic (I-L) and advanced stage (M-P) of the disease. In the first line boxes, a schematic representation of the L2 coronal section is shown, with a grey square showing the white matter portion analyzed: vmWM (A, E, I, M), vlWM (B, F, J, N), dlWM (C, G, K, O) and dWM (D, H, L, P). Scale bar 50 μm.
(TIF)
