# Supplementary figures and images for: Comparative Magnetic Resonance Imaging and Histopathological Correlates in Two SOD1 Transgenic Mouse Models of Amyotrophic Lateral Sclerosis
Source: PLoS One. 2015 Jul 1;10(7):e0132159. doi: 10.1371/journal.pone.0132159 (PMC4488470; doi:10.1371/journal.pone.0132159)

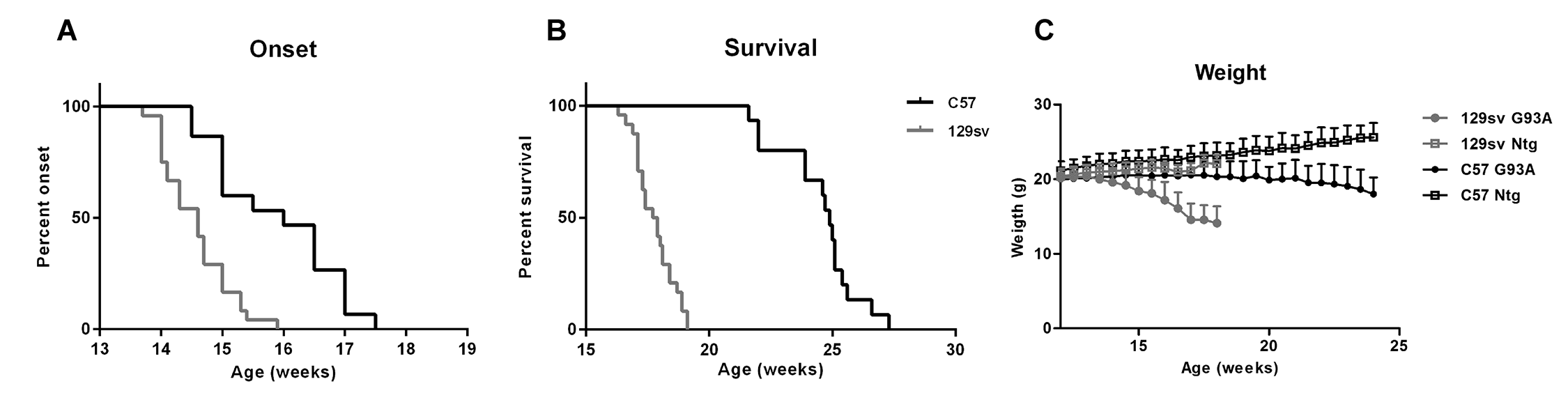

Supplement: S1 Fig — Kaplan Meier plots showing the proportion of transgenic mice without symptoms of disease onset (a) and percentage of surviving animals (b) are reported. From graphs it is possible to appreciate the earlier onset and the faster disease progression in 129Sv SOD1G93A mice, compared to C57 SOD1G93A mice. Indeed, C57 SOD1G93A mice displayed first motor symptoms at 15.9 ±1 weeks of age (mean body weight 20.5g ± 1.7) and died at 24.5 ±1.6 weeks (mean body weight 18g ± 1.7), while 129Sv SOD1G93A mice show the onset of motor impairment at 14.5± 0.6 weeks (mean body weight 17.2g ± 2.5) and they died at 17.8 ±0.8 weeks of age (mean body weight 14.1g ± 2.2). The statistical analysis by the Log-rank test reveals a p-value < 0.0001 for both onset and survival. (c) Weight change of C57 and 129Sv SOD1G93A mice during the disease progressio. Data are expressed as mean ± SD. N = 15 animals for C57 SOD1G93A mice and 24 animals for 129Sv SOD1G93A mice. (TIF) [file pone.0132159.s001.tif]

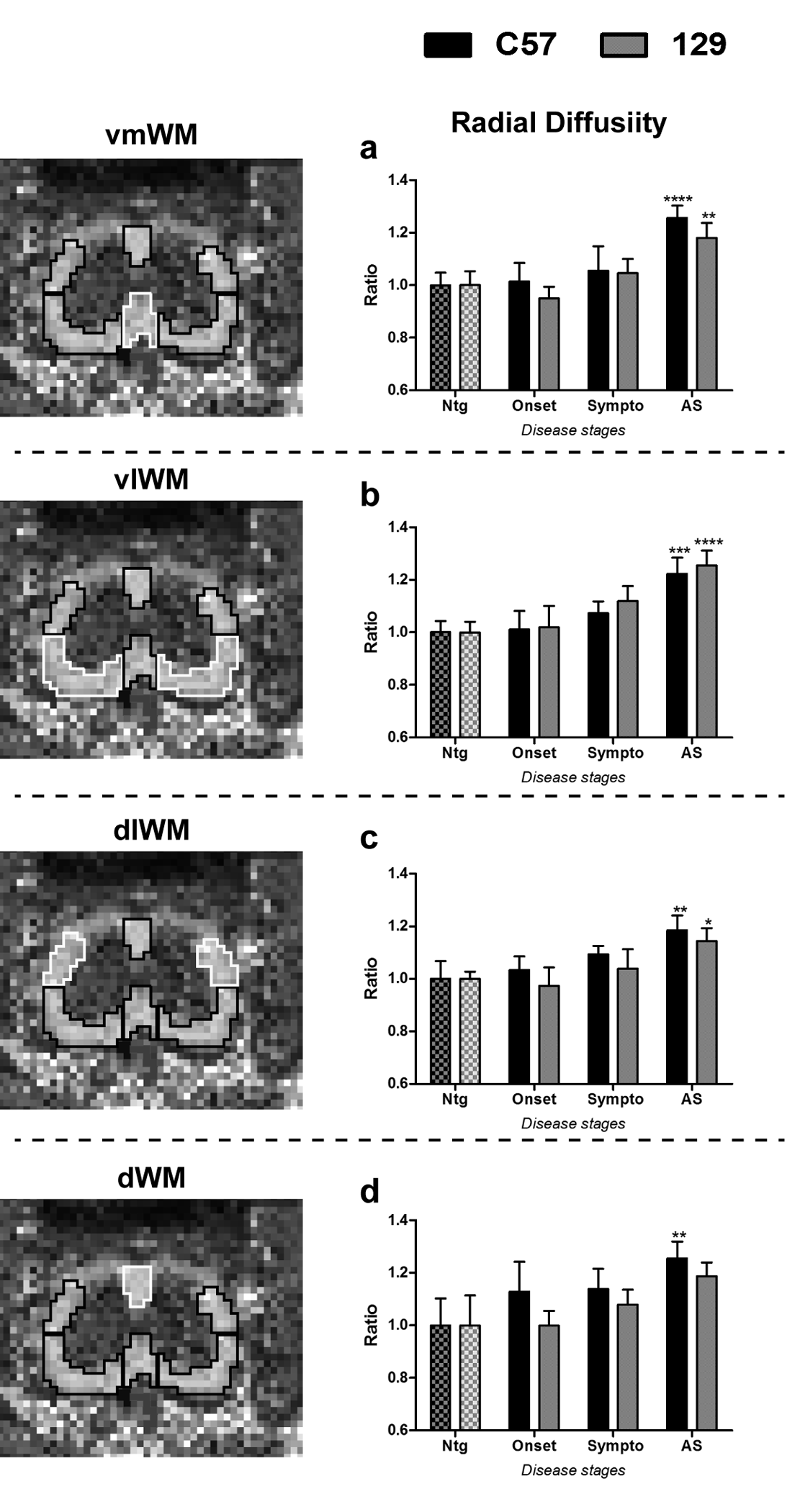

Supplement: S2 Fig — Radial diffusivity of different white matter regions has been reported: ventro-medial (vmWM, a), ventro-lateral (vlWM, b), dorso-lateral (dlWM, c) and dorsal (dWM, d). On the left, representative images of the Fractional Anisotropy of the lumbar spinal cord have been reported. The white line represents the white matter area taken into consideration. (TIF) [file pone.0132159.s002.tif]

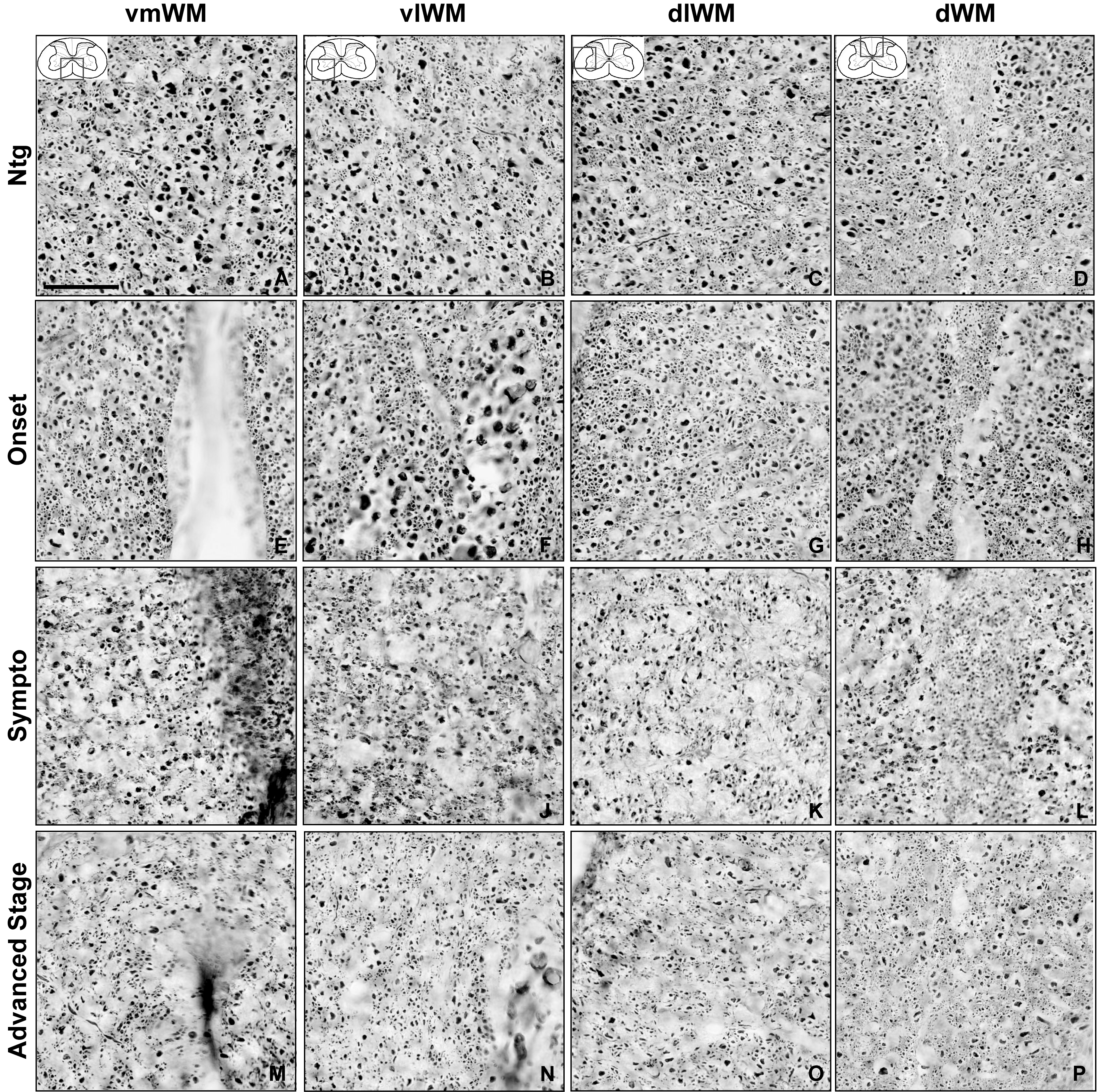

Supplement: S3 Fig — Microphotographs of SMI-31 staining of the lumbar white matter spinal cord are reported from a non-transgenic (A-D) and C57 SOD1G93A mice at the onset (E-H), symptomatic (I-L) and advanced stage (M-P) of the disease. In the first line boxes, a schematic representation of the L2 coronal section is shown, with a grey square showing the white matter portion analysed: vmWM (A, E, I, M), vlWM (B, F, J, N), dlWM (C, G, K, O) and dWM (D, H, L, P). Scale bar 50 μm. (TIF) [file pone.0132159.s003.tif]

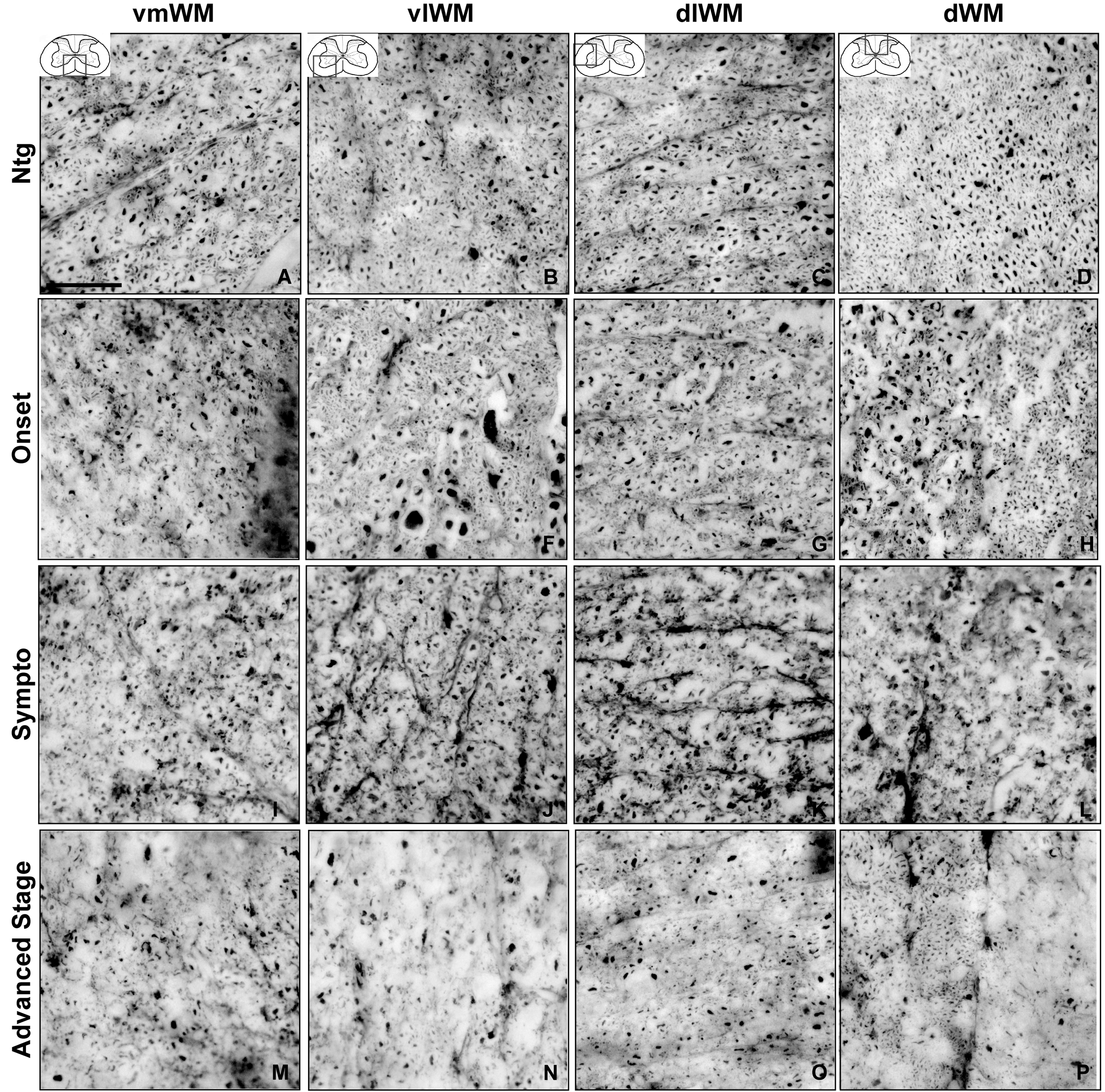

Supplement: S4 Fig — Microphotographs of SMI-31 staining of the lumbar white matter are reported from a non-transgenic (A-D) and 129Sv SOD1G93A mice at the onset (E-H), symptomatic (I-L) and advanced stage (M-P) of the disease. In the first line boxes, a schematic representation of the L2 coronal section is shown, with a grey square showing the white matter portion analyzed: vmWM (A, E, I, M), vlWM (B, F, J, N), dlWM (C, G, K, O) and dWM (D, H, L, P). Scale bar 50 μm. (TIF) [file pone.0132159.s004.tif]
